# The predictors of general knowledge: Data from a Spanish megastudy

**DOI:** 10.3758/s13428-021-01669-4

**Published:** 2021-08-06

**Authors:** Francisco Buades-Sitjar, Roger Boada, Marc Guasch, Pilar Ferré, José Antonio Hinojosa, Jon Andoni Duñabeitia

**Affiliations:** 1 Centro de Investigación Nebrija en Cognición, Universidad Antonio de Nebrija, Santa Cruz de Marcenado 27, 28015 Madrid, Spain; 2 Department of Psychology and CRAMC, Tarragona, Spain; 3 Instituto Pluridisciplinar, Universidad Complutense de Madrid, Madrid, Spain; 4 Dpto. Psicología Experimental, Procesos Cognitivos y Logopedia, Universidad Complutense de Madrid, Madrid, Spain; 5 Department of Languages and Culture, The Arctic University of Norway, Tromsø, Norway

**Keywords:** General knowledge, Intelligence, Cultural knowledge, Sociodemographic data, Trivia quiz

## Abstract

Studies on sociodemographic data and crystallized intelligence have often struggled to recruit enough participants to achieve sufficient validity. However, the advent of the internet now allows this problem to be solved through the creation of megastudies. Yet, this methodology so far has only been used in studies on vocabulary size, while general knowledge, another key component of crystallized intelligence, remains unexamined. In the present study, regression models were used to examine the impact of sociodemographic variables—gender, age, years of study and socioeconomic status—on general knowledge scores. The sample comprised 48,234 participants, each of whom answered 60 general knowledge questions, their data being fully available online. Men were found to score higher than women in general knowledge. Years of study and socioeconomic status acted as strong and weak positive predictors, respectively. Age acted as a strong positive predictor until the age of 50, where it became progressively detrimental. These results are discussed relative to other studies on crystallized intelligence, highlighting the need to study each of its components individually.

Intelligence is a construct of a paradoxical nature. On one hand, it is an extremely intuitive concept, with most people having some sort of internalized idea of what constitutes "being intelligent." On the other, when asked to put such idea into words, most people would be hard-pressed to find an accurate definition that completely encapsulates what intelligence is, and said definition would likely vary from individual to individual. Such a predicament is also true within the scientific community, as decades upon decades of debate and research have been invested in finding ways to properly define and measure intelligence (see Kaufman et al., 2013, for a review), and yet multiple caveats on the matter remain unsolved.

One idea that is usually agreed upon, though, is that intelligence can be divided into two major constructs: fluid intelligence (i.e., cognitive processes not subservient to prior knowledge, such as pattern recognition, reasoning and abstraction); and crystallized intelligence (i.e., declarative and procedural knowledge learnt by an individual throughout their life span, such as verbal ability or general knowledge) (Ackerman et al., 2001). This distinction is of critical importance, as each type of intelligence has been proven to be beneficial in different manners. For instance, fluid intelligence is able to predict academic success at earlier life stages, while crystalized intelligence predicts academic and professional success at later stages (Ackerman, 1996). Another example of this division is the fact that, in decision-making tasks, fluid intelligence increases an individual's resistance to framing effects and facilitates the application of newly established rules, while crystalized intelligence protects against the influence of sunken costs (Bruine de Bruin, 2012).

However, it is crucial to keep in mind that crystallized and fluid intelligence are not completely independent from each other, but rather interact in critically meaningful ways. For instance, crystalized intelligence is considered to be the result of investing fluid intelligence in the process of learning and acquiring new knowledge and skills (Chamorro-Premuzic et al., 2005). Indeed, it would make sense that a strong ability to draw associations and finding patterns would make it easier to store and retrieve information from one's memory. In a similar fashion, crystallized knowledge born from experience can open up new tools and ways of thinking with which to tackle typically fluid problems (Taub et al., 2008).

Given the unique benefits that both crystallized and fluid intelligence bring to the table, it is unsurprising that many resources have been invested in trying to understand how they are specifically influenced by individual variables. When it comes to personality measures, while both types of intelligence are predicted by the openness and emotional stability factors (Chamorro-Premuzic et al., 2005; Furnham & Chamorro-Premuzic, 2006), this relationship seems to be much stronger for crystalized than for fluid intelligence (Rammstedt et al., 2018). In regard to sociodemographic variables, fluid intelligence has been shown to decrease with age after early adulthood (Bugg et al., 2006), while crystallized intelligence reaches its maximum at 65 years of age and remains stable after that (Bowles & Salthouse, 2008; Singh-Manoux, 2012).

Nevertheless, when it comes to crystallized intelligence, there is one critical caveat to be kept in mind. Any test attempting to measure such construct would require a huge number of items in order to try to capture the entire breadth of an individual's knowledge. This, in turn, necessitates the use of proportionally large sample sizes in order to gather enough trials per item to achieve solid validity. Fortunately, the widespread use of the internet has provided us with a tool to solve this problem thanks to the so-called *megastudies*. These massive experiments can take advantage of the far-reaching power of the internet to draw data from thousands of participants, provided only that the task can be performed online with no experimenter supervision. This opens up the possibility of creating databases with gigantic numbers of items that not only equal, but often far surpass, the number of trials per item of traditional experiments. One example of this is the Programme for the International Assessment of Adult Competencies, or PIAAC (OECD, 2013), a cyclic megastudy that evaluates the literacy and numeracy skills of people between 16 and 65 years of age across multiple countries. These skills are evaluated by asking participants to read texts and answer questions about them, completing sentences with the most appropriate ending, solving mathematical problems or interpreting charts and graphs. Such tasks require a certain level of reasoning and abstraction, thus involving a fluid component, but they also require a variety of previously acquired knowledge, being largely based on crystallized intelligence. Other experimental approaches exploring certain aspects of crystalized intelligence are nicely represented in the studies by Brysbaert et al. (2016) and Aguasvivas et al. (2020), which gathered data on vocabulary size through lexical decision tasks^1^. One major contribution of these studies is demonstrating that vocabulary size does not decrease, or reach a plateau, after 65 years of age, as other studies had previously claimed (Bowles & Salthouse, 2008; Singh-Manoux, 2012), but rather continues to increase throughout an individual's life span. This is somewhat at odds with the findings of the PIAAC, showing that the skills measured reach their peak at early adulthood and are maintained until about the age of 35, from which point they start to progressively decline. However, one point in common between the studies by Brysbaert et al. (2016) and Aguasvivas et al. (2020) and the PIAAC studies is that they all found that men had a slight tendency to obtain higher scores than women.

These studies, however, do not cover general knowledge, considered a key aspect of crystallized intelligence defined as the ability to retrieve culturally relevant facts and data from memory. The skills measured by the PIAAC are mainly procedural, in contrast to the strictly declarative nature of general knowledge. Meanwhile, vocabulary size does not consider either proper nouns or whether participants know the meaning of the words, both being key aspects of general knowledge. Therefore, considering that general knowledge relies on clearly distinct factors of crystallized intelligence, it is critical that its particularities be separately studied.

Yet, while much effort has been put into creating scales to measure an individual's general knowledge level—the most prominent ones being Nelson and Narens (1980) and Tauber et al. (2013), but see also Duñabeitia et al. (2016), Jalbert et al. (2019) and Martín-Luengo et al. (2020)—most of the research in which they have been employed has not focused on such constructs per se. Instead, these scales have mostly been utilized as a source of questions and statements in experiments studying phenomena such as illusory truth (Fazio et al., 2015), metacognition (Jackson & Greene, 2014; Weinstein & Roedinger, 2010) and error correction (Sitzman et al., 2014; Sitzman et al., 2015), as the wide range of topics they cover acts as an easy way to control for the influence of the content of the items.

Still, some key studies can be found specifically examining general knowledge. With regard to its particular relationship with intelligence, Chamorro-Premuzic et al. (2005) found that crystallized intelligence predicted up to 30% of the variance in general knowledge scores, while fluid intelligence predicted about 10%. This not only confirms the already intuitive notion that general knowledge is a subset of crystallized intelligence, but also supports the idea that crystallized intelligence is ultimately the product of investing fluid intelligence in the process of learning. When it comes to its relationship with individual variables, specific personality factors have been linked to general knowledge. For instance, while the positive effect that openness has on crystallized intelligence is also present in general knowledge, some studies report no positive correlation with emotional stability (Chamorro-Premuzic et al., 2005; Furnham & Chamorro-Premuzic, 2006). Finally, when examining sociodemographic variables, Beier and Ackerman (2001) found moderate positive correlations between general knowledge scores and both educational level and age, although the strength of the latter was significantly influenced by the specific field of knowledge of the items. Coane and Umanath (2012) also found evidence pointing to the beneficial effect of age on general knowledge, showing that older adults now obtained higher scores on the Nelson and Narens (1980) questionnaire compared to the young adults that comprised the original sample. Ackerman et al. (2001) also found that men tend to outperform women in general knowledge tasks, but that these differences were heavily mediated by non-ability factors such as confidence and personality measures. In the same vein, a study by Steinmayr et al. (2015) suggested that gender differences are partially driven by factors unrelated to general knowledge per se. They found that men and women with the same general knowledge levels had different probabilities of correctly answering some items, an effect known as differential item functioning (DIF).

Despite the strong methodology of these studies, they are still gated by the same limitations as the traditional studies on vocabulary size. Hence, in the present megastudy, we examine how four key sociodemographic variables—gender, age, years of study and socioeconomic status—influence individuals' general knowledge level. To this end, a large-scale study was carried out with a set of 1270 general knowledge questions extracted from Buades-Sitjar et al. (2021) that were tested in a trivia quiz in which nearly 85,000 Spanish individuals participated. While this study is exploratory in nature and does not attempt to prove one specific hypothesis, we do venture to make some predictions on the results based on previous research. In particular, we expect to obtain similar results as those of Beier and Ackerman (2001), where age and years of study are significant predictors of general knowledge scores, with the latter being a stronger, more consistent predictor.

## Method

### Participants

**Fig. 1:**
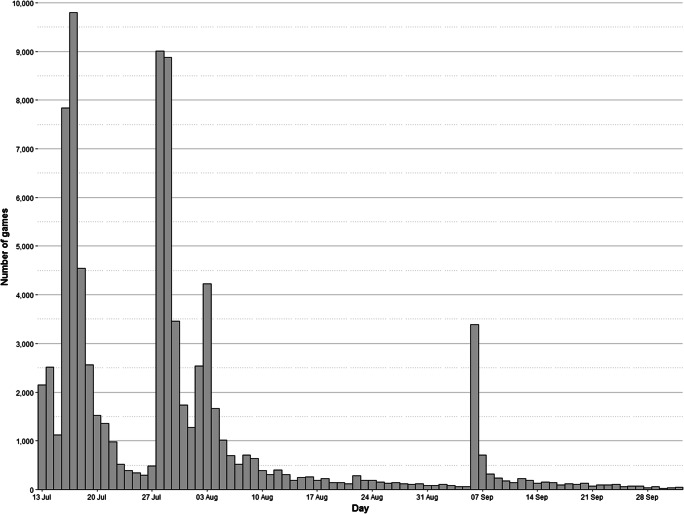
Distribution of the number of games played each day throughout the entire data collection period

**Fig. 2:**
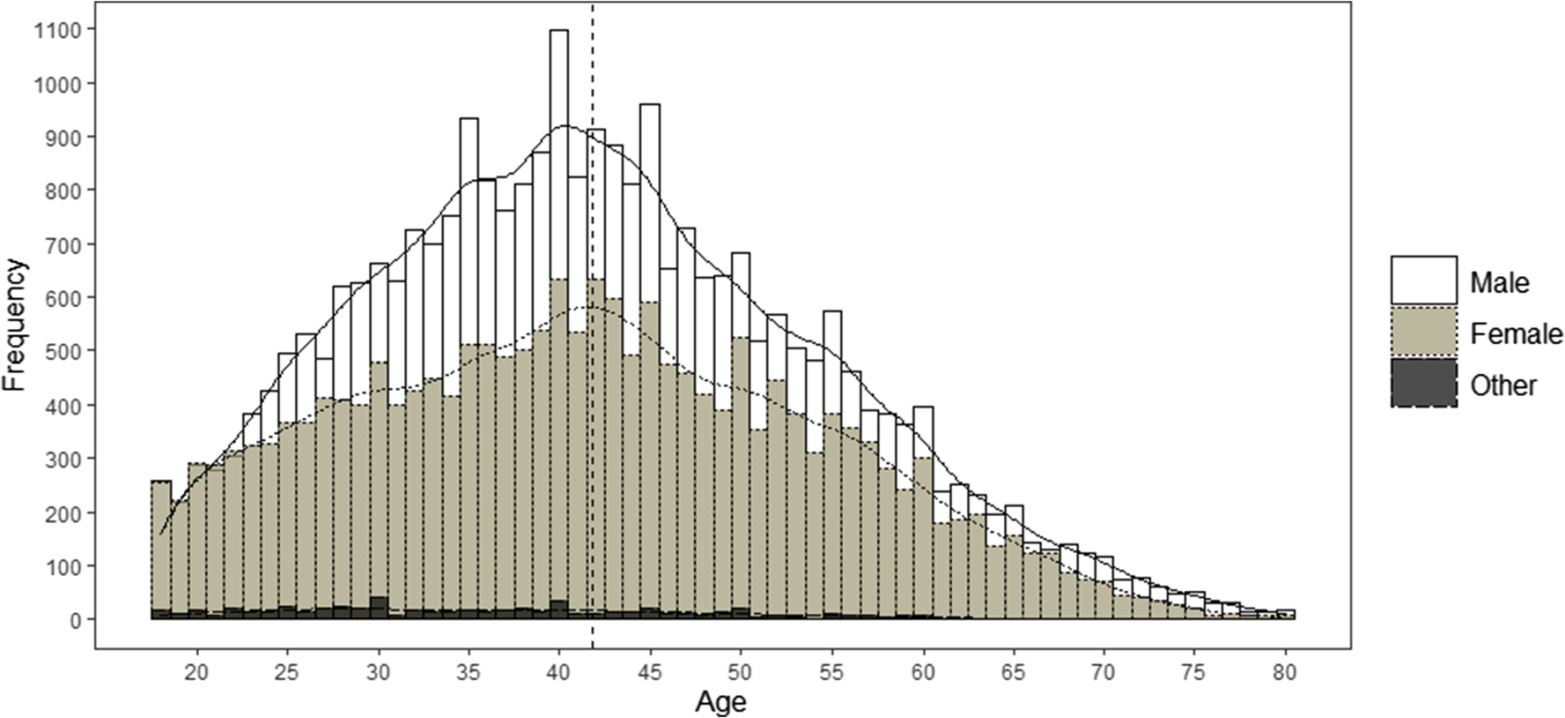
Distribution and density plots of the number of games played by age. Data are clustered by gender, with each bar stacked in front of each other. The vertical dashed line marks the mean age of the total sample

The web platform used for data collection was launched on July 13, 2020, and it was closed on October 2, 2020. During these 99 days, a total of 84,613 games were played. The platform was initially disseminated through social networks, but since participants were able to share their results through their own networks, i.e., Facebook, Twitter, WhatsApp, Telegram, and email, a snowball effect soon took place. A dissemination campaign was carried out at the same time by mass media at the provincial and national level, i.e., online and written press, internet forums, radio programs, etc. The competitive and viral effect that the questionnaire achieved meant that it reached people from a wide range of backgrounds, ensuring that it did not attract only people with a natural intellectual curiosity. Furthermore, the simple procedure of the game only required basic technological skills to participate, which 98% of the Spanish population claims to have (Instituto Nacional de Estadística, 2019). Figure 1 shows the number of games played per day during the entire data collection period. The peaks that are observed throughout the period correspond to the specific moments when the study was broadcast in the press or on the radio as a means of participants’ recruitment. The distributions by gender and age of the participants are displayed in Fig. 2.

**Fig. 3:**
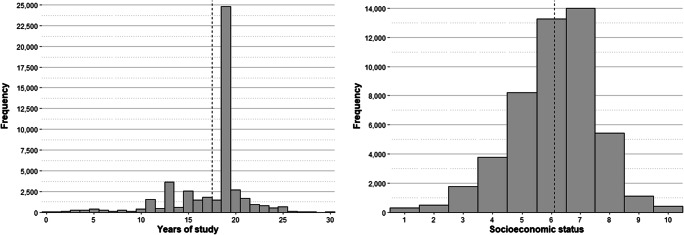
Distribution of the years of study (left) and the socioeconomic status (right) reported by the participants. Vertical dashed lines indicate the mean value of each variable

Among the participants, 58.56% were men, 40.14% were women and 1.30% did not identify as either men or women^2^. Regarding age distribution, the mean age of the participants was 41.66 years (SD = 12.60, range [18–80]). Concerning sociodemographic data, participants were asked to indicate the number of years spent in official studies and their socioeconomic level, assessed on a scale of 1 to 10 (see the “Procedure” section below for more information). The distribution of both variables is shown in Fig. 3. The mean years of study reported by the participants was 17.46 years (SD = 3.90, range [0–25]), while the average socioeconomic level was 6.11 (SD = 1.48, range [1–10]). Regarding the first variable, more than half of the sample is concentrated around the value of 19. This is the number of years that Spanish citizens who have a university degree spend in official studies.

### Materials

A subset of 1270 items were extracted from Buades-Sitjar et al. (2021), a general knowledge question database with over 1350 items covering 37 different fields of knowledge (e.g., art, history, philosophy, technology, biology, etc.). Each of the items includes a general knowledge question (e.g., "*What is the name of the organ that produces insulin?*") and four answer options (e.g., *"Pancreas," "Kidney," "Spleen"* and *"Bladder"*). A list of the different fields of knowledge that comprise the database can be found in Table 1, along with the number of visualizations per category and a sample question. The database includes the pick-rate for the correct answer and for each of the incorrect options, as well as a link to a Wikipedia article in which the answer can be checked. The subset of questions used in this study was selected based on their difficulty—namely, excluding questions with a hit-rate showing highest or lowest accuracy—and their atemporality—namely, whether the ratings for the question were likely to change over time. Finally, we only selected items whose question length did not exceed 100 characters and whose answer length did not exceed 50 characters, as it would be inconvenient for the format they were presented in (see “Procedure”).

**Table 1:** Number of visualizations and sample questions for each field of knowledge

Category	# of visualizations	Question example
Architecture	84578	What architectural style does Big Ben belong to?
Art	89413	To which art movement did Frida Kahlo belong?
Astronauts	60080	When was the International Space Station launched into space?
Astronomy	89444	What is the Latin name for the North Star?
Biology	82916	What are multicellular organisms that have an even number of chromosomes called?
Botany	87349	What name is given to the plants whose seeds are encased inside a fruit?
Brands	55133	Who were, along with Steve Jobs, the co-founders of Apple?
Chemistry	87251	What is table salt also known as?
Classical music	85293	Who composed the "Ode to Joy," the official anthem of the European Union?
Economy	78271	What is the term for a business or a company run by a single person?
Europe	78370	What is the longest river in Europe?
Food	73823	What is the animal whose milk is used to make Mozzarella cheese?
Geography	87037	Which of these is a tropical grassland?
History	94488	When did the American Civil War take place?
Human body	80091	How often is the epidermis renewed with a new crop of cells?
Inventions	87575	What invention is attributed to Galileo Galilei?
Linguistics	92464	What do you call a language that is created artificially instead of naturally?
Literature	80257	Which science fiction writer wrote the three laws of robotics?
Math	61815	How many degrees does the sum of the angles of a regular pentagon add up to?
Measurements	80255	How many horses is a kilowatt?
Medicine	92135	What is the medical term for a low blood sugar level?
Movies	71117	Who directed movies like "Titanic," "Terminator" 1 and 2, "Aliens" and "Avatar"?
Music	78421	Which singer is known for the single "My Heart Will Go On"?
Mythology	121755	What animal breastfed Romulus and Remus according to Roman mythology?
Organizations	59853	Where are the headquarters of the World Health Organization?
Philosophy	73883	What famous philosophical work includes the Allegory of the Cave?
Physics	82402	What color of visible light has the shortest wavelength?
Politics	87170	What is the Fifth Amendment to the United States Constitution also known as?
Psychology	82682	What is the scientific name for photographic memory?
Records	47964	What is the most cultivated fruit tree in the world?
Religion	91740	What is the last book of the Bible?
Sports	68728	What is the tennis term that denotes that each side has a score of 40 in a game?
Technology	76172	What does the acronym "RAM" mean in computing?
Television	25027	What is Marge Simpson's maiden name?
Transports	63881	What electronic device ensures that the fuel/air mixture in a car engine is correct?
World	87066	What was the previous name of the island of Sri Lanka?
Zoology	91601	How many chambers comprise the stomach of a cow?

Table 2 displays the results of the linear regression, in which gender, age, years of study and socioeconomic status were used as predictors of general knowledge scores. A significant regression equation was found (*F*_(6,48226)_ = 1557, *p* < 0.001), with an *R*^2^ of 0.164. The data show that men (β = 6.13, SE = 0.09) and people who did not identify as either men or women (β = 6.23, SE = 0.40) tended to score higher in general knowledge than women (Intercept). As suggested by visual inspection of the data, age proved to have a quadratic-like relationship with general knowledge scores: it acted as a positive predictor before the age of 50 (β = 0.29, SE = 0.05), but became steadily more detrimental after that (β = −0.17, SE = 0.01). Education was also a solid predictor, increasing the mean scores by 0.56 (SE = 0.01) per each year of study. Finally, socioeconomic status acted as a weak predictor, increasing scores by 0.16 (SE = 0.01) per level in the scale. All predictors, as well as the intercept, were significant (*p* < .001) (see Fig. 5).

### Procedure

The data were collected through a web application accessible at the following URL: https://lagranpregunta.es/. The first page displayed a general information section about the study, the contact address of the authors and the number of games played so far. To further encourage engagement, we also included participation maps and the ability to share the study on social media. Participants provided their consent to participate in the study by clicking on the "continue" button.

On the next page, sociodemographic data were collected on age, gender, nationality, autonomous community, province of residence and number of years spent studying (only official studies had to be reported). Participants also had to indicate whether it was their first time participating in the study. Before starting the experiment, participants were asked to rate their socioeconomic status on a scale of 1 to 10 by using the MacArthur Scale of Subjective Social Status (Adler & Stewart, 2007).

Finally, participants were provided with the instructions for the game, which were as follows: “*You will be presented with 60 questions on different topics with 4 answer options. You must indicate what you think is the correct answer before the 15 seconds time limit expires. If you take longer, the answer to that question will be computed as an error. Try to answer all the questions! When you finish, we will inform you about the percentage of questions you got right and about your performance in comparison with the other participants.”*

Once the game started, participants were presented with the questions one by one, for a total of 60 questions. The questions were randomly chosen from the 1270-item pool, and the order of presentation on the screen of the four answer options was randomized across participants. Participants who played the game on a PC provided their answers by clicking on them, while those who played the game on mobile devices did so by tapping on the screen. A blue bar at the top of the screen marked the progress of the game. If participants did not respond within a 15-second period, a pop-up encouraged them to respond more quickly, and that question was skipped.

After completing the test, a thank-you page informed the participant about the percentage of correct answers. Participants were also informed of the percentile of their score in relation to all the games played so far, and could see a map of Spain displaying the average scores by autonomous community. At the bottom of the screen, the participant’s performance was detailed, with a list containing all the correct, omitted and failed questions. Each item included the correct answer and a link to a Wikipedia page with information on the question subject. A series of buttons allowed participants to share their score on social networks, i.e., Facebook, Twitter, WhatsApp, Telegram and email. There was also a link that enabled participants to access detailed information on the purpose of the study. Finally, two buttons allowed participants either to begin another game without having to fill in the sociodemographic data again, or to start another game as a different user.

### Data analysis

Participants could take the test as many times as they wished, but the main goal of this study was to characterize the general level of knowledge while avoiding the effect of practice and the possible appearance of repeated questions. Because of this, only the first game played by each participant—i.e., unique participants—was selected for the final analyses. This resulted in the removal of 34.53% of the total data. Moreover, we also removed those games with an anomalous response pattern and those not properly recorded due to technical errors—e.g., data with empty responses or with values outside the allowed range, impossible response times. As a consequence, 3.01% of the total data were eliminated. In addition, those participants who indicated that their nationality was not Spanish (3.57% of the data) were also removed, since the interest of the study is the assessment of the general knowledge of the Spanish population. We also removed all participants who claimed to have studied for longer than 25 years, as they covered an excessively wide range (26 to 52 years of study) in spite of representing only 0.47% of the data, which could bias our results. Finally, an age filter was also applied, discarding participants older than 80 years, as the number of participants above that age was marginal and therefore not very representative (0.08% of data). Participants who did not reach the legal age to participate without parental consent were also removed (1.34% of the data). Thus, the final sample analyzed included 48,234 games made by unique participants, for a total of 2,894,040 individual item responses (see Fig. 2 in the participants section for the distribution by sex and age).

**Table 2:** Estimated fixed effects of predictors for general knowledge scores

				95% CI	
Parameter	β	SE	t	Lower	Upper
Intercept	39.71	0.10	389.22	−50.11	−50.62
Gender male	6.13	0.09	65.85	5.94	6.31
Gender other	6.23	0.40	15.40	5.43	7.02
Age ≤ 50	0.29	0.005	53.90	0.28	0.30
Age > 50	−0.17	0.01	14.23	−0.20	−0.14
Years of study	0.56	0.01	46.90	0.54	0.58
SES	0.16	0.03	4.97	0.09	0.23

*Note.* The predictor *Age ≤ 50* is obtained by subtracting 18 from the participant's age, and has a maximum value of 50. The predictor *Age > 50* is obtained by subtracting 50 from the participant's age, and has a value of 0 for those under 50 years old.

In order to analyze the predictive power of each sociodemographic variable on the participants' scores, two types of regression analyses were performed using R (R Core Team, 2020). The first analysis consisted of a multiple linear regression in which the hit rate of each participant—i.e., their percentage of correct responses—was predicted based on their gender, age, years of study and socioeconomic status (SES). Visual inspection of the scatterplots for each predictor variable and the dependent variable revealed a quadratic-like, inverted U-shape relationship between the age of participants and their scores. To account for this nonlinear relationship, a linear spline (degree = 1) with a knot at Age = 50 was used by calling the *bs* function from the *splines* package. The regression was computed using the *lm* function and the formula *rate ~ gender + age_spline + years of study + SES*.

The second analysis consisted of a logistic mixed effects model used to predict the chances of correctly answering a random item from the questionnaire. To that end, each individual response to an item—i.e., 60 items × 48,234 participants = 2,894,040 individual responses—was matched with the gender, age, years of study and SES of the participant who responded to it. These variables were used as the fixed effects, while the participant ID and the item ID were used as random effects. The model was computed using the *glmer* function from the R package *lmer4* (Bates et al., 2015) and the following formula: *hit ~ gender + age_spline + years of study + SES + (1| id_user) + (1| id_item)*. The age spline was calculated using the same method as in the linear regression.

The data corresponding to the analyzed sample can be accessed as supplementary material to this article. They include three csv files: “items.csv,” which contains all the information related to the 1270 items—i.e., their identifier, category, the main text of the question, the four response options texts and the number of occurrences; “users.csv,” which contains the information related to the 48,234 participants—i.e., their identifier, their number of hits, start and end time of the game and the sociodemographic data included in the initial questionnaire; and “answers.csv,” which contains the information of the 2,894,040 responses from the participants in each question—i.e., their identifier, order of appearance of the item in the game, the selected answer, whether said answer was correct or not and response time. The three files can be linked to each other through the corresponding identification fields of item, user and response—"id_item," "id_user," and "id_trial," respectively. The supplementary material can be accessed via 10.6084/m9.figshare.14073899

## Results

**Fig. 4:**
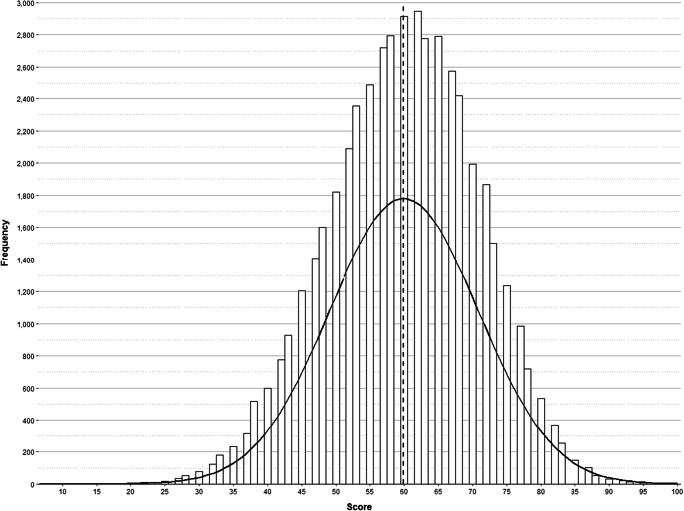
Distribution of the scores. The dashed line corresponds to the statistical mean

First, we examined the distribution of the scores obtained by participants (see Fig. 4), computed as the percentage of hits—i.e., number of hits divided by number of questions. The average score was 59.93% (SD = 10.90, range [6.67–100]). Regarding the items, each question was seen by an average number of 2297 participants (SD = 47.46, range [2150–2430]).

**Fig. 5:**
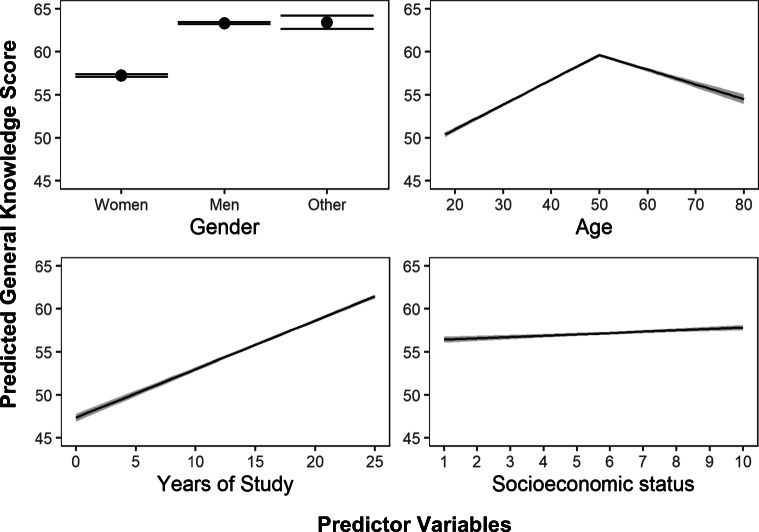
Plotted regressions of predicted general knowledge scores

**Table 3:** Estimated fixed effects of predictors for chances of correctly answering a random item

					95% CI	
Predictor	β	SE	Z	OR	Lower	Upper
Intercept	−0.571	0.437	1.30	0.564	−1.496	0.131
Gender male	0.359	0.005	69.84	1.431	0.349	0.369
Gender other	0.365	0.022	16.43	1.440	0.321	0.408
Age ≤ 50	0.016	<0.001	56.52	1.016	0.016	0.016
Age >50	−0.009	<0.001	15.40	0.991	−0.010	−0.008
Years of study	0.032	<0.001	49.62	1.032	0.031	0.033
SES	0.009	<0.001	5.30	1.009	0.006	0.012

*Note.* The predictor *Age ≤50* equals the age of the participant, and has a maximum value of 50. The predictor *Age > 50* equals is obtained by subtracting 50 from the participant's age, and has a value of 0 for those under 50 years old.

**Fig. 6:**
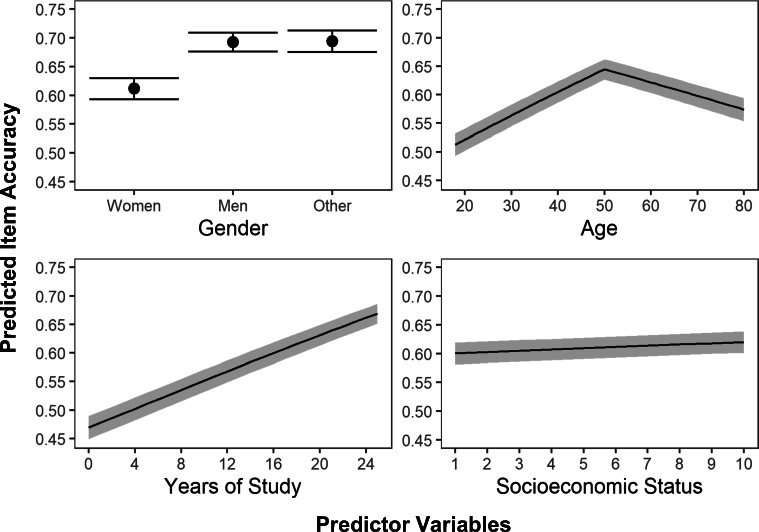
Plotted regressions of predicted item accuracy

Table 3 displays the results of the model used to predict the chances of an individual correctly answering a random item based on their gender, age, years of study and socioeconomic status. The model successfully converged, explaining 42% of the variance. The variance explained by the fixed effects was 1.2% (*p* < .001), and the variance explained by the random effects (participants and items) was 40.8% (*p* < .001). In a similar fashion to the previous model, men (β = 0.359, SE = 0.005, OR = 1.431) and people who did not identify as either men or women (β = 0.365, SE = 0.022, OR = 1.440) had higher chances of correctly answering a random question than women did. The same quadratic-like relationship with age was also found, acting as a positive predictor before the age of 50 (β = 0.016, SE < 0.001, OR = 1.016) but slowly becoming detrimental after that threshold (β = −0.009, SE < 0.001, OR = 0.991). Finally, both years of study (β = 0.032, SE < 0.001, OR = 1.032) and socioeconomic status (β = 0.009, SE < 0.001, OR = 1.009) increased the chances of correctly answering a random item from the test, with the former being a stronger predictor than the latter. All predictors were statistically significant (*p* < .001), but the intercept was not (*p* = 0.19) (see Fig. 6).

To ensure that the gender differences in our results were not caused by differential item functioning—a possibility pointed out by Steinmayr et al. (2015)—we ran an additional exploratory analysis using the *difLogistic* function from the R package *difR.* This function uses logistic regressions in order to examine whether individuals from different subgroups—in this case, male and female—with the same underlying ability, i.e., same general knowledge scores, have different chances of giving a correct response to an item. The analysis concluded that 643 of the 1270 items displayed a statistically significant differential item functioning, as expected from such a large sample size, but that the effect sizes were so abysmally small as to be considered negligible (Nagelkerke's *R*^2^ = 0.001–0.01).

## Discussion

In order to capture the entirety of a person's knowledge, studies examining the influence between sociodemographic variables and crystallized intelligence require the use of questionnaires with an incredibly large number or items. This, in turn, also requires monumentally large sample sizes in order to achieve proper validity, which so far has been considered as excessively time consuming. However, the advent of the internet has provided a way to bypass this issue, allowing one to easily gather data from thousands of participants at a time. Nevertheless, these megastudies have yet to examine a specific subcomponent of crystallized intelligence: general knowledge. Therefore, in this megastudy we investigated how sociodemographic variables such as gender, age, years of study and socioeconomic status influence a person's general knowledge.

Our results reveal a quadratic-like relationship between the age of participants and their general knowledge scores, as well as their chances of correctly answering a random item from the questionnaire. The analyses indicate that age acts as a positive influence for general knowledge up to middle age—around 50 years old—after which it becomes progressively detrimental. This finding contrasts with similar megastudies examining vocabulary size, which found that age consistently acted as a positive predictor throughout a person’s life span (Aguasvivas et al., 2020; Brysbaert et al., 2016), and numeracy and literacy skills, which were found to peak at early to mid-adulthood (OECD, 2013). Hence, our results highlight the need for a specialized study of each component of crystalized intelligence.

We hypothesize that the differential influence of age on vocabulary size and general knowledge lies in the very nature of these two constructs. Vocabulary size only requires the ability to correctly recognize a string of letters as a word, without needing to be able to produce a definition or use it in context. General knowledge, however, requires the recollection of definitions, proper nouns, numbers and dates, as well as highly specific technical words, being an overall much more declarative type of construct. Older adults are usually better equipped in general knowledge than younger cohorts, and in fact tend to rely more on previous knowledge to compensate for their deficits in fluid intelligence (Umanath & Marsh, 2014). However, while the knowledge required to answer a question might be stored in memory, older adults are also more prone to retrieval failures of declarative information (Cantor et al., 2015). In fact, previous research shows that older adults tend to rely on gist-like knowledge instead of explicitly declarative knowledge (Castel et al., 2007; Koutstaal and Schacter, 1997; Paige et al., 2016). While the multiple-choice format of our questions should help by turning the task into one of recognition, instead of retrieval, it still requires accessing very specific declarative information. Therefore, when prompted with questions such as "*What biblical story describes the creation of the different languages?*" older adults might remember that the general plot of the story explains how a group of people who wanted to build a tower to reach God were punished by making them speak different languages. However, they might not remember that the passage was specifically called "*The Tower of Babel.*"^3^

In a related vein, when considering the differential influence of age on literacy and numeracy skills and general knowledge, the different components that the two types of tests tap into should be taken into account. The significant fluid component of the PIAAC tasks may put older people at a disadvantage, as this type of intelligence tends to peak at early to mid-adulthood (Bugg et al., 2006), which is precisely where the PIAAC scores begin to decline. In contrast, the declarative nature of the current general knowledge test mainly requires that participants rely on crystallized intelligence, so that only after 50 years, when retrieval difficulties start to appear, does age become detrimental.

Years of study also proved to be a solid positive predictor of general knowledge scores and the chances of correctly answering a random item. These results are unsurprising, as there is already a solid body of research showcasing the positive impact of years of educational on intelligence scores. In particular, a meta-analysis conducted by Ritchie and Tucker-Drob (2018) shows that IQ scores increase by about two points per year of education, and that years of education is the strongest predictor of such scores. The novelty of our study is that it examines the influence of education in a very specific sub-construct of intelligence—general knowledge—and that it indicates that age acts as a stronger positive influence than years of education, at least up until middle age, where age starts becoming detrimental while years of study remains linearly positive.

Socioeconomic status proved to be a weak predictor for general knowledge. In fact, due to its low impact and large confidence intervals, it would be reasonable to even dismiss it as predictor altogether, even in spite of it being significant. This finding contrasts with previous studies indicating that socioeconomic status exerts a considerable positive influence on IQ scores (Hanscombe et al., 2012; Von Stumm & Plomin, 2015), highlighting again the need for independent and specialized study of each of the subcomponents of intelligence. Of note is that the studies cited here examine the relationship between socioeconomic status and IQ in younger populations, while our sample consisted exclusively of people over 18 years of age. Therefore, it is possible that the influence of socioeconomic status is more prevalent during the early years, while slowly smoothing during adulthood. Furthermore, it should be kept in mind that the socioeconomic status of the younger portion of the sample—under 30 years of age—is more likely to be dependent on the socioeconomic status of their parents, rather than their own status per se. Hence, caution is advised when interpreting these 35w?>In regard to gender, our results indicate that men tended to obtain higher scores than women in general knowledge and they had higher chances of correctly answering a random item. These results align with those found in megastudies on vocabulary size, literacy and numeracy, where men have been found to perform better than women (see Aguasvivas et al., 2020; OECD; 2013). Our results also align with previous research on general knowledge, such as Ackerman (2001), showing that men tend to perform better than women on general knowledge tasks. Steinmayr et al. (2015) found evidence that these differences could be partially due to differential item functioning. However, while a similar differential item functioning was also found here, the effect sizes were so small as to be considered negligible. Hence, the exact nature of these gender differences remains to be explored, as it seems unlikely that they are purely biological in origin. In fact, certain studies have found that gender differences in literacy and numeracy greatly vary with age and across countries and cultures (Borgonovi, Choi, & Paccagnella, 2021; Chiu & McBride-Chang, 2006). Future studies should be aimed at examining which societal and demographic components might be boosting these differences, paying special attention to developmental and territorial factors.

The current study highlights that different aspects of crystallized intelligence should be studied differentially. Together with studies on vocabulary size and literacy and numeracy skills, this study reveals how sociodemographic variables exert different influences in all types of crystallized intelligence. Therefore, similarly to how fluid intelligence has been typically divided into its various subcomponents, our study underlines the critical importance of following the same process with crystallized intelligence. Future research should attempt to break down the construct of crystallized intelligence into all its possible subcomponents, so that they can be studied considering their specific nuances. Furthermore, the interaction between fluid and crystallized intelligence—and their respective subcomponents—should also be examined. So far, these two types of intelligence have mostly been studied independently from one another, but as previous research has pointed out, these two systems work in a cooperative fashion (Chamorro-Premuzic et al., 2005; Taub et al., 2008).

In conclusion, this study presents the first large-scale general knowledge database obtained from a large sample of Spanish native speakers, and it offers evidence of the modulating impact of different sociodemographic factors on general cultural knowledge. All the data are made available to the scientific community to facilitate investigating the role of different modulating variables and to give rise to new scientific studies and international collaborations that help to strengthen our understanding of the concept of general knowledge in society.

## Footnotes

1 An extensive list of similar studies can be found at http://crr.ugent.be/programs-data/megastudy-data-available

2 While our sample contains a higher percentage of men than the one in the Spanish population (~49%; Instituto Nacional de Estadística, 2020), the large sample size ensures that the number of men and women is sufficiently high as to draw an accurate picture of the general Spanish citizenship.

3 One could argue that the detrimental effect of age after 50 years old might be caused by people over 50 having lower education levels. Indeed there is a small negative correlation between age and years of study in our sample (−0.15). However, after running an independent regression using only the participants over the age of 50, we found that the negative effect of age after 50 years old was still present.

## References

[CR1] Ackerman PL (1996). A theory of adult intellectual development: Process, personality, interests, and knowledge. Intelligence.

[CR2] Ackerman PL, Bowen KR, Beier ME, Kanfer R (2001). Determinants of individual differences and gender differences in knowledge. Journal of Educational Psychology.

[CR3] Adler, N., & Stewart, J. (2007). The MacArthur scale of subjective social status. MacArthur Research Network on SES & Health. http://www.macses.ucsf.edu/Research/Psychosocial/subjective.php.

[CR4] Aguasvivas, J., Carreiras, M., Brysbaert, M., Mandera, P., Keuleers, E., & Duñabeitia, J. A. (2020). How do Spanish speakers read words? Insights from a crowdsourced lexical decision megastudy. *Behavior Research Methods*, 1-16. 10.3758/s13428-020-01357-9 10.3758/s13428-020-01357-9 32072567

[CR5] Bates D, Maechler D, Bolker B, Walker S (2015). Fitting Linear Mixed-Effects Models Using lme4. Journal of Statistical Software.

[CR6] Beier ME, Ackerman PL (2001). Current-events knowledge in adults: An investigation of age, intelligence, and nonability determinants. Psychology and Aging.

[CR7] Bowles RP, Salthouse TA (2008). Vocabulary test format and differential relations to age. Psychology and Aging.

[CR8] Borgonovi F, Choi Á, Paccagnella M (2021). The evolution of gender gaps in numeracy and literacy between childhood and young adulthood. Economics of Education Review.

[CR9] Bruine de Bruin W, Parker AM, Fischhoff B (2012). Explaining adult age differences in decision-making competence. Journal of Behavioral Decision Making.

[CR10] Brysbaert M, Stevens M, Mandera P, Keuleers E (2016). How many words do we know? Practical estimates of vocabulary size dependent on word definition, the degree of language input and the participant’s age. Frontiers in psychology.

[CR11] Buades-Sitjar, F., Boada, R., Guasch, M., Ferré, P., Antonio Hinojosa, J., Brysbaert, M., & Andoni Duñabeitia, J. (2021). The thousand-question Spanish general knowledge database. *Psicológica, 42*(1). 10.2478/psicolj-2021-0006

[CR12] Bugg JM, Zook NA, DeLosh EL, Davalos DB, Davis HP (2006). Age differences in fluid intelligence: contributions of general slowing and frontal decline. Brain and cognition.

[CR13] Castel AD, Farb NA, Craik FI (2007). Memory for general and specific value information in younger and older adults: Measuring the limits of strategic control. Memory & Cognition.

[CR14] Cantor AD, Eslick AN, Marsh EJ, Bjork RA, Bjork EL (2015). Multiple-choice tests stabilize access to marginal knowledge. Memory & Cognition.

[CR15] Chamorro-Premuzic T, Moutafi J, Furnham A (2005). The relationship between personality traits, subjectively-assessed and fluid intelligence. Personality and Individual Differences.

[CR16] Chiu MM, McBride-Chang C (2006). Gender, context, and reading: A comparison of students in 43 countries. Scientific studies of reading.

[CR17] Coane, J. H., & Umanath, S. A. (2012). Database of general knowledge question performance in older adults. *Behavior Research Methods*, 1-15. 10.3758/s13428-020-01493-2 10.3758/s13428-020-01493-2 PMC7880974 33443730

[CR18] Duñabeitia JA, Griffin KL, Martín JL, Oliva M, Sámano ML, Ivaz L (2016). The Spanish General Knowledge Norms. Frontiers in psychology.

[CR19] Fazio LK, Brashier NM, Payne BK, Marsh EJ (2015). Knowledge does not protect against illusory truth. Journal of Experimental Psychology: General.

[CR20] Furnham A, Chamorro-Premuzic T (2006). Personality, intelligence and general knowledge. Learning and Individual Differences.

[CR21] Hanscombe KB, Trzaskowski M, Haworth CM, Davis OS, Dale PS, Plomin R (2012). Socioeconomic status (SES) and children's intelligence (IQ): In a UK-representative sample SES moderates the environmental, not genetic, effect on IQ. PloS one.

[CR22] Instituto Nacional de Estadística (2019). *Encuesta sobre Equipamiento y Uso de Tecnologías de Información y Comunicación en los Hogares*[Data File]. https://www.ine.es/prensa/tich_2019.pdf

[CR23] Instituto Nacional de Estadística (2020). *Cifras de Población (CP) a 1 de julio de 2020* [Data File]. https://www.ine.es/prensa/cp_j2020_p.pdf

[CR24] Jackson A, Greene RL (2014). Impression formation of tests: Retrospective judgments of performance are higher when easier questions come first. Memory & Cognition.

[CR25] Jalbert, M., Newman, E., & Schwarz, N. (2019). Trivia claim norming: Methods report and data. *ResearchGate*.10.6084/m9.figshare.9975602

[CR26] Kaufman, J. C., Kaufman, S. B., & Plucker, J. A. (2013). Contemporary theories of intelligence. *The Oxford Handbook of Cognitive Psychology*, 811-822. 10.1093/oxfordhb/9780195376746.013.0051

[CR27] Koutstaal W, Schacter DL (1997). Gist-based false recognition of pictures in older and younger adults. Journal of memory and language.

[CR28] Martín-Luengo B, Zinchenko O, Alekseeva M, Shtyrov Y, Y. (2020). Russian norms for 500 general-knowledge questions. Front. Psychology.

[CR29] Nelson TO, Narens L (1980). Norms of 300 general-information questions: Accuracy of recall, latency of recall, and feeling-of-knowing ratings. Journal of Verbal Learning and Verbal Behavior.

[CR30] OECD (2013). PIAAC Data Explorer. https://piaacdataexplorer.oecd.org/ide/idepiaac/

[CR31] Paige LE, Cassidy BS, Schacter DL, Gutchess AH (2016). Age differences in hippocampal activation during gist-based false recognition. Neurobiology of aging.

[CR32] R Core Team (2020). R: A language and environment for statistical computing. R Foundation for Statistical Computing, Vienna, Austria. https://www.R-project.org/.

[CR33] Rammstedt B, Lechner CM, Danner D (2018). Relationships between personality and cognitive ability: A facet-level analysis. Journal of Intelligence.

[CR34] Ritchie, S. J., & Tucker-Drob, E. M. (2018). How much does education improve intelligence? A meta-analysis. *Psychological Science*, *29*(8), 1358-1369. 10.1177/0956797618774253 10.1177/0956797618774253 PMC6088505 29911926

[CR35] Singh-Manoux, A., Kivimaki, M., Glymour, M. M., Elbaz, A., Berr, C., Ebmeier, K. P., ... & Dugravot, A. (2012). Timing of onset of cognitive decline: results from Whitehall II prospective cohort study. *Bmj, 344*, d7622. 10.1136/bmj.d7622 10.1136/bmj.d7622 PMC3281313 22223828

[CR36] Sitzman DM, Rhodes MG, Tauber SK (2014). Prior knowledge is more predictive of error correction than subjective confidence. Memory & Cognition.

[CR37] Sitzman DM, Rhodes MG, Tauber SK, Liceralde VRT (2015). The role of prior knowledge in error correction for younger and older adults. Aging, Neuropsychology, and Cognition.

[CR38] Steinmayr R, Bergold S, Margraf-Stiksrud J, Freund PA (2015). Gender differences on general knowledge tests: Are they due to Differential Item Functioning?. Intelligence.

[CR39] Taub GE, Keith TZ, Floyd RG, McGrew KS (2008). Effects of general and broad cognitive abilities on mathematics achievement. School Psychology Quarterly.

[CR40] Tauber, S. K., Dunlosky, J., Rawson, K. A., Rhodes, M. G., & Sitzman, D. M. (2013). General knowledge norms: Updated and expanded from the Nelson and Narens (1980) norms. *Behavior Research Methods, 45(4)*, 1115-1143. 10.3758/s13428-012-0307-9 10.3758/s13428-012-0307-9 23344739

[CR41] Umanath, S., & Marsh, E. J. (2014). Understanding how prior knowledge influences memory in older adults. *Perspectives on Psychological Science*, *9*(4), 408-426. 10.1177/1745691614535933 10.1177/1745691614535933 26173273

[CR42] Von Stumm S, Plomin R (2015). Socioeconomic status and the growth of intelligence from infancy through adolescence. Intelligence.

[CR43] Weinstein Y, Roediger HL (2010). Retrospective bias in test performance: Providing easy items at the beginning of a test makes students believe they did better on it. Memory & Cognition.

